# Worldwide distribution of *NAT2 *diversity: Implications for *NAT2 *evolutionary history

**DOI:** 10.1186/1471-2156-9-21

**Published:** 2008-02-27

**Authors:** Audrey Sabbagh, André Langaney, Pierre Darlu, Nathalie Gérard, Rajagopal Krishnamoorthy, Estella S Poloni

**Affiliations:** 1 INSERM U535, BP 1000, 94817 Villejuif Cedex, France; 2 Laboratory of Genetics and Biometry, Department of Anthropology and Ecology, University of Geneva, Geneva, Switzerland; 3 Muséum National d'Histoire Naturelle, Paris, France; 4 INSERM U763, Paris, France

## Abstract

**Background:**

The N-acetyltransferase 2 (*NAT2*) gene plays a crucial role in the metabolism of many drugs and xenobiotics. As it represents a likely target of population-specific selection pressures, we fully sequenced the *NAT2 *coding region in 97 Mandenka individuals from Senegal, and compared these sequences to extant data on other African populations. The Mandenka data were further included in a worldwide dataset composed of 41 published population samples (6,727 individuals) from four continental regions that were adequately genotyped for all common *NAT2 *variants so as to provide further insights into the worldwide haplotype diversity and population structure at *NAT2*.

**Results:**

The sequencing analysis of the *NAT2 *gene in the Mandenka sample revealed twelve polymorphic sites in the coding exon (two of which are newly identified mutations, C345T and C638T), defining 16 haplotypes. High diversity and no molecular signal of departure from neutrality were observed in this West African sample. On the basis of the worldwide genotyping survey dataset, we found a strong genetic structure differentiating East Asians from both Europeans and sub-Saharan Africans. This pattern could result from region- or population-specific selective pressures acting at this locus, as further suggested in the HapMap data by extremely high values of *F*_ST _for a few SNPs positions in the *NAT2 *coding exon (T341C, C481T and A803G) in comparison to the empirical distribution of *F*_ST _values accross the whole 400-kb region of the *NAT *gene family.

**Conclusion:**

Patterns of sequence variation at *NAT2 *are consistent with selective neutrality in all sub-Saharan African populations investigated, whereas the high level of population differentiation between Europeans and East Asians inferred from SNPs could suggest population-specific selective pressures acting at this locus, probably caused by differences in diet or exposure to other environmental signals.

## Background

The N-acetyltransferase 2 (*NAT2*) gene plays a crucial role in the metabolism of xenobiotics, including many clinically useful drugs and exogenous chemicals present in the diet, cigarette smoke and the environment [1]. Extensive polymorphism in *NAT2 *gives rise to a wide interindividual variation in N-acetylation capacity. In particular, a clear bimodal distribution is observed that segregates the rapid acetylator phenotype, associated with a normal acetylation capacity, from the slow acetylator one, characterized by a reduced enzyme activity. These two main metabolic phenotypes occur with varying prevalence in populations of different ethnic origin [2].

The clinical consequences of the acetylation polymorphism can be severe if standard drug doses are applied, exposing patients to an increased risk of adverse drug reactions or a lack of therapeutic efficacy [3]. In addition, in the last decades, an increasing number of epidemiological studies have attempted to relate acetylation phenotype to a variety of complex human disorders, such as bladder cancer, atopic diseases, diabetes, Parkinson's disease and many others (see Butcher et al. [4] for a review). However, up to now, association studies in *NAT2 *have led to conflicting results among (and even within) human populations and most association findings have been difficult to replicate. One reason for these inconsistencies may relate to the fact that almost all studies focused on a limited number of candidate polymorphisms, which were not necessarily the same from one study to another [5,6]. A shift toward a gene-based approach in which all common variation within a gene is considered jointly is advocated for future association studies [7]. By capturing all of the potential risk-conferring variations within *NAT2*, this approach should resolve much of the controversial issues of candidate-polymorphism studies.

There is now a large body of information on the distribution of *NAT2 *genetic variants all over the world [8]. However, most published reports used simplified protocols for *NAT2 *allele detection, omitting analysis of several polymorphic positions (such as G191A, C282T, T341C, and A803G within the coding region). They focused on a limited number of "indicator" mutations, thought to be tightly linked with other mutations and predictive of acetylator status, and have based allele designation on this. Such incomplete genotyping methods wrongly type different alleles as the same and may lead to misclassification of genotypes and deduced phenotypes [9]. Therefore, results of such investigations may be substantially biased and fail to provide an accurate picture of *NAT2 *allele distribution in worldwide populations.

In an attempt to better characterize the worldwide haplotype diversity and LD structure of *NAT2*, we performed an extensive survey of the literature to identify those samples that were adequately genotyped for all common variants in *NAT2*. In total, 41 population samples (including 6,727 individuals) from four continental regions (Africa, Europe, Asia, America) were selected and jointly analyzed. In addition, we performed full sequencing analysis of the *NAT2 *coding region in a large and ethnically well defined Mandenka sample from Eastern Senegal so as to further characterize African diversity at this locus and to detect novel variants not yet reported. Beyond a simple description of *NAT2 *gene diversity, the goal of the present study was to provide further insights into the evolutionary forces that most likely shaped *NAT2 *genetic variation in humans. In particular, three levels of diversity (intrapopulation, interpopulation, and interspecies variability) were used to investigate to what extent present *NAT2 *variation patterns solely reflect stochastic events of human evolution, or are distorted by natural selection.

## Results

### *NAT2* sequence diversity in the Mandenka and variation in Sub-Saharan Africa

Results of the sequencing analysis of *NAT2 *in the Mandenka sample are reported in Table 1. A total of 12 polymorphic sites were identified, all located within the *NAT2 *coding exon. Two of them were singletons confirmed by resequencing. Apart from the seven SNPs that are commonly found in human populations, we found three additional variants that have been recently reported by Patin et al. [10] in several sub-Saharan African samples (C403G, G609T, G838A), and two novel nucleotide changes not yet described: C345T and C638T, which occurred at a frequency of 0.026 and 0.01, respectively. C638T leads to an amino acid change (P213L). Sixteen distinct haplotypes were inferred by the PHASE program, including two which were recently described in the Patin et al. data set [10] and four that are newly described here. Among these six haplotypes, four contain inactivating mutations and are thus predicted as 'slow alleles', whereas the other two (NAT2*12g and NAT2*12H) contain a nonsynonymous mutation (G609T or C403G) whose impact on phenotype is unknown. From diplotype configurations at *NAT2 *in the Mandenka, we inferred 48.5%, 39.2%, and 7.2% of slow, intermediate, and rapid acetylators, respectively. The remaining 5.1% individuals had an unknown acetylator status as they were carriers of either a NAT2*12g or a NAT2*12H haplotype.

**Table 1:** SNP and haplotype frequencies in the Mandenka sample.

Nucleotide Change^a^	G191A	C282T	T341C	C345T^d^	C403G^c^	C481T	G590A	G609T^c^	C638T^d^	A803G	G838A^c^	G857A	
Amino Acid Change	R64Q	None	I114T	None	L135V	None	R197Q	E203D	P213L	K268R	V280M	G286E	
SNP Frequency	0.1031	0.3041	0.3608	0.0258	0.0206	0.3454	0.1701	0.0051	0.0103	0.5052	0.0051	0.0722	
Haplotype^b^													Haplotype Frequency
NAT2*4^e^	G	C	T	C	C	C	G	G	C	A	G	G	0.0928
NAT2*5A	.	.	C	.	.	T	.	.	.	.	.	.	0.0103
NAT2*5B	.	.	C	.	.	T	.	.	.	G	.	.	0.3300
NAT2*5C	.	.	C	.	.	.	.	.	.	G	.	.	0.0155
NAT2*6A	.	T	.	.	.	.	A	.	.	.	.	.	0.1289
NAT2*7B	.	T	.	.	.	.	.	.	.	.	.	A	0.0670
NAT2*12A	.	.	.	.	.	.	.	.	.	G	.	.	0.1289
NAT2*13	.	T	.	.	.	.	.	.	.	.	.	.	0.0515
NAT2*14A	A	.	.	.	.	.	.	.	.	.	.	.	0.0876
NAT2*14B	A	T	.	.	.	.	.	.	.	.	.	.	0.0155
NAT2*12g^c^	.	.	.	.	.	.	.	T	.	G	.	.	0.0052
NAT2*6J^d^	.	T	.	.	.	.	A	.	.	.	.	A	0.0052
NAT2*12H^c^	.	.	.	.	G	.	.	.	.	G	.	.	0.0206
NAT2*5M^d^	.	.	C	.	.	T	.	.	.	G	A	.	0.0052
NAT2*6K^d^	.	T	.	.	.	.	A	.	T	.	.	.	0.0103
NAT2*6L^d^	.	T	.	T	.	.	A	.	.	.	.	.	0.0258
**2N**^f^													**194**

^a ^Polymorphic sites were numbered considering +1 as the A of the translation start codon in the cDNA sequence (GenBank CR407631)

^b ^*NAT2 *haplotypes were named in accordance with the consensus gene nomenclature of human *NAT2 *alleles [65]

^c ^These SNPs/haplotypes have been recently described in Patin et al. [10]

^d ^Newly reported SNPs/haplotypes in the present study

^e ^NAT2*4 is the reference allele that was shown to be the ancestral haplotype through outgroup comparisons with the chimpanzee and rhesus monkey sequences

^f ^Total number of chromosomes in the Mandenka sample

Summary statistics of genetic variation at the *NAT2 *coding region (870 bp) in the Mandenka and the 12 African samples of Patin et al. [10] are reported in Table 2. Results for the entire surveyed region (1188 bp) in the Mandenka are also indicated. Patterns of diversity in this sample are entirely consistent with those displayed by the 12 other African samples. The mean values of the two nucleotide-variability measures, *π *and *θ*_*w*_, for the 13 samples are 0.268% and 0.221%, respectively. None of the tests of selective neutrality, performed on each sample both at the intrapopulation and at the interspecies levels, yielded significant results (not shown). This suggests that patterns of diversity at *NAT2 *are consistent with the hypothesis of selective neutrality and constant population size. The average sequence divergence between human and chimpanzee for *NAT2 *coding exon was of 1.6%, and the average substitution rate of 1.6 × 10^-9 ^per nucleotide and year. All samples provided similar estimates both for *Ne*, the current effective population size, and *T*_MRCA_, the coalescence time back to the most recent common ancestor: average values were 14,218 individuals and 1.077 My, respectively, for the *NAT2 *coding exon. The age of mutations in the gene genealogy ranged from 96,200 (G857A) to 496,300 (A803G) years for the seven SNPs that commonly occur in human populations. The other polymorphisms, only reported to date in sub-Saharan Africans, had all estimated ages < 50,000 years.

**Table 2:** Summary statistics and coalescence estimates for *NAT2 *sequence data.

Sample	***H***^a^	***π***^b^	***θ***_*w*_^c^	***Ne***^d^	***T*_*MRCA*_**^e^
Mandenka (1188 bp)	0.837	0.218	0.173	10,932	799 ± 267
Mandenka (870 bp)	0.837	0.298	0.238	15,658	1,145 ± 382
12 African populations^f ^(870 bp)					
*mean*^g^	0.806	0.265	0.220	14,098	1,071 ± 372
*standard deviation*^g^	± 0.061	± 0.038	± 0.034	± 2,733	
*minimum*^g^	0.678	0.181	0.156	9,173	947 ± 332
*maximum*^g^	0.879	0.302	0.271	19,903	1,297 ± 418

^a ^Haplotype diversity

^b ^Nucleotide diversity per bp (× 10^-2^) with Jukes and Cantor's correction

^c ^Watterson's estimator of the population mutation parameter per bp (× 10^-2^)

^d ^Effective population size

^e ^Time to the most recent common ancestor (*T*_MRCA_) of all sampled genes (in thousand years)

^f ^Sequence data from Patin et al. [10]

^g ^Are reported here the mean (and standard deviation), minimum, and maximum values of the statistics computed for each of the twelve sub-Saharan African samples of Patin et al. [10]

### *NAT2* worldwide genotyping survey

A total of 6,727 individuals from 41 worldwide samples were analyzed for their genotype at the seven common SNPs of the *NAT2 *gene. All SNPs and populations were in Hardy-Weinberg equilibrium after Bonferroni correction for multiple testing. The seven SNPs defined 21 distinct haplotypes, whose composition in terms of SNP variants is given in Table 3.

**Table 3:** *NAT2 *haplotype^a ^frequencies in samples of the worldwide genotyping survey^b^.

	*NAT2 *Haplotype^e^																					
	*4	*5A	*5B	*5C	*5D	*5E	*5M	*6A	*6B	*6C	*7A	*7B	*11A	*12A	*12B	*12C	*13	*13B	*14A	*14B	*6J^f^	**Global (2N^g^)**
SNP position^c ^(ancestral state^d^)																						
191 (G)	.	.	.	.	.	.	.	.	.	.	.	.	.	.	.	.	.	.	**A**	**A**	.	
282 (C)	.	.	.	.	.	.	T	T	.	T	.	T	.	.	T	.	T	T	.	T	T	
341 (T)	.	**C**	**C**	**C**	**C**	**C**	**C**	.	.	.	.	.	.	.	.	.	.	.	.	.	.	
481 (C)	.	T	T	.	.	.	.	.	.	.	.	.	T	.	.	T	.	T	.	.	.	
590 (G)	.	.	.	.	.	**A**	.	**A**	**A**	**A**	.	.	.	.	.	.	.	.	.	.	**A**	
803 (A)	.	.	G	G	.	.	.	.	.	G	.	.	.	G	G	G	.	.	.	.	.	
857 (G)	.	.	.	.	.	.	.	.	.	.	**A**	**A**	.	.	.	.	.	.	.	.	**A**	

Population code^h^																						
1	27	1	47	17	0	0	0	34	4	0	0	0	0	34	7	1	13	0	6	11	0	**202**
2	10	1	37	3	0	0	1	16	0	0	0	2	0	9	2	0	6	0	3	10	0	**100**
3	8	0	4	0	0	0	0	11	0	0	0	0	0	39	1	0	13	0	0	4	0	**80**
4	4	0	13	5	0	0	0	8	0	0	0	0	0	19	3	0	6	0	0	2	0	**60**
6	18	2	65	3	0	0	0	32	0	0	0	13	0	30	0	0	10	0	17	3	1	**194**
7	13	0	22	8	0	0	0	32	5	0	2	1	0	7	1	0	4	0	2	3	0	**100**
8	3	0	19	0	0	0	0	17	0	0	0	1	0	7	0	0	0	0	1	0	0	**48**
9	13	0	45	0	0	0	0	22	0	0	0	3	0	4	0	0	1	0	0	0	0	**88**
10	133	1	242	0	0	0	0	127	2	0	0	3	0	6	0	0	0	0	2	0	0	**516**
11	20	1	52	1	0	0	0	24	0	0	0	0	0	0	0	0	0	0	0	0	0	**98**
12	22	6	54	2	0	0	0	30	0	0	0	1	0	0	0	0	5	0	0	0	0	**120**
14	44	8	104	6	0	0	0	54	1	0	0	5	0	1	0	0	1	0	0	0	0	**224**
15	187	20	318	17	0	0	0	206	0	0	0	15	0	3	0	0	7	0	0	1	0	**774**
17	11	6	50	3	0	0	0	28	0	0	0	2	0	0	0	0	0	0	0	0	0	**100**
18	23	3	42	0	0	0	0	15	0	0	0	13	0	0	0	0	0	0	0	0	0	**96**
19	383	70	647	68	0	0	0	470	0	0	0	22	0	0	0	0	26	0	0	2	0	**1688**
21	109	26	164	30	0	0	0	149	0	0	0	17	0	1	0	0	0	0	0	0	0	**496**
23	9	0	41	0	0	0	0	29	0	0	0	1	0	0	0	0	0	0	0	0	0	**80**
25	134	12	213	17	0	0	0	184	0	0	0	17	0	3	0	0	0	0	0	0	0	**580**
26	140	8	216	29	0	0	0	185	0	0	0	27	0	1	0	0	0	0	0	0	0	**606**
27	13	1	29	4	0	0	0	43	0	1	0	6	0	3	0	0	0	0	0	0	0	**100**
28	31	0	23	3	0	0	0	30	0	0	0	12	0	1	0	0	0	0	0	0	0	**100**
30	132	0	4	0	1	0	0	46	0	0	0	38	0	0	0	0	3	0	0	0	0	**224**
32	46	0	6	0	0	0	0	22	0	0	0	13	0	0	0	0	1	0	0	0	0	**88**
33	208	0	1	0	0	1	0	55	0	0	0	22	0	0	0	0	1	0	0	0	0	**288**
36	353	1	5	0	1	0	0	126	3	0	6	70	0	4	0	1	6	0	0	0	0	**576**
38	26	0	10	0	0	0	0	34	0	0	0	17	0	0	0	0	1	0	0	0	0	**88**
41	114	7	86	5	0	0	0	46	1	1	0	0	0	8	0	2	3	0	1	0	0	**274**
Global	2234	174	2559	221	2	1	1	2075	16	2	8	321	0	180	14	4	107	0	32	36	1	**7988**

^a ^*NAT2 *haplotypes were named in accordance with the consensus gene nomenclature of human *NAT2 *alleles [65]

^b ^Only the 28 samples with no missing data (i.e., with genotype data available for the seven common SNPs in *NAT2*) were considered here

^c ^Polymorphic sites were numbered considering +1 as the A of the translation start codon in the cDNA sequence (GenBank CR407631)

^d ^The ancestral state of each SNP was deduced from both the chimpanzee and rhesus monkey sequences, and is represented here as a dot

^e ^Nucleotide substitutions shown in bold have a functional consequence on enzyme activity. Bold-faced haplotypes are 'slow alleles' associated with as decreased acetylation capacity; the others display an enzymatic activity comparable to the reference 'rapid' allele NAT2*4

^f ^NAT2*6J is a newly described allele found in the Mandenka sample in the present study (see Table 1). It was predicted to be a 'slow allele' since it contains two inactivating mutations

^g ^Total number of chromosomes

^h ^Samples are numbered according to their population code [see Additional file 1]: (1) 101 Tswana, (2) 50 Ateke Bantus, (3) 40 Bakola Pygmies, (4) 30 Baka Pygmies, (6) 97 Mandenka, (7) 50 Dogons, (8) 24 Somali, (9) 44 Moroccans, (10) 258 Spanish, (11) 49 Sardinians, (12) 60 French, (14) 112 UK Caucasians, (15) 387 US Caucasians, (17) 50 Swedes, (18) 48 Saami, (19) 844 Germans, (21) 248 Polish, (23) 40 Ashkenazi Jews, (25) 290 Russians, (26) 303 Turks, (27) 50 Gujarati, (28) 50 Turkmen, (30) 112 Han Chinese, (32) 44 Chinese, (33) 144 Japanese, (36) 288 Koreans, (38) 44 Thai, (41) 137 Nicaraguans.

#### Continental distribution of *NAT2* SNP variants and haplotypes

**Figure 1:**
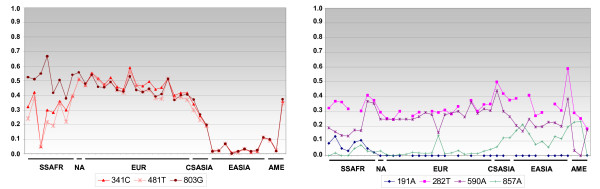
**SNP frequencies in the 41 samples of the worldwide genotyping survey.** Samples are ordered geographically (as in Additional file 1), thus including: SSAFR, sub-Saharan Africa (Tswana, Ateke Bantus, Bakola Pygmies, Baka Pygmies, Yoruba, Mandenka, Dogons, and Somali); NA, North Africa (Moroccans); EUR, Europe (Spanish, Sardinians, French, French-Canadians, UK Caucasians, both US Caucasian samples, Swedes, Saami, both German samples, Polish, Slovaks, Ashkenazi Jews, Romanians, Russians, and Turks); CSASIA, Central/South Asia (Gujarati, Turkmen, and Kyrgyz); EASIA, East Asia (the three Chinese, three Japanese, and two Korean samples, and Thai); AME, America (Embera, Ngawbe, and Nicaraguans).

**Figure 2:**
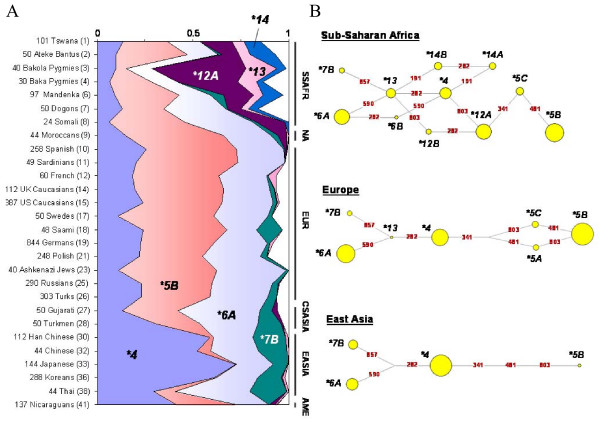
**(A) Haplotype frequencies in the 28 samples genotyped for all seven common SNPs at *NAT2 *(i.e., without missing data), taken from the worldwide genotyping survey.** Single populations are reported on the left side of the plot, with population codes in brackets; geographic areas are indicated on the right side, as follows: SSAFR, sub-Saharan Africa; NA, North Africa; EUR, Europe; CSASIA, Central/South Asia; EASIA, East Asia; AME, America. Only haplotypes with frequencies > 5% in at least one geographical region were represented individually; all other haplotypes were pooled into a single group (in white). Also, haplotypes NAT2*14A and NAT2*14B were pooled into the NAT2*14 cluster. **(B) Median-joining networks of the inferred *NAT2 *haplotypes within three geographical regions: sub-Saharan Africa, Europe, and East Asia.** Only haplotypes with frequencies > 0.005 within a geographic area were considered to construct the networks. Circle areas are proportional to the haplotypes' frequency, and branch lengths are proportional to the number of mutations separating haplotypes. Haplotypes' labels are shown in black; mutations are shown in red on corresponding network branches.

Allele frequency variation of the seven common SNPs of *NAT2 *in the 41 sampled populations is shown in Figure 1, and the worldwide distribution of common *NAT2 *haplotypes is displayed in Figure 2A. Figure 1 clearly highlights the high correlation in frequency of the three variants 341C, 481T, and 803G in Europe, Asia and America. This reflects the high level of LD between these three SNPs which, in combination, form the NAT2*5B haplotype. This variant occurs at a high frequency in Europe but is rare in populations of Asian origin. In sub-Saharan Africa, a poorer correlation between 803G and the two other variants is observed. This can be explained by the higher frequency of NAT2*12A in Africa, in which the 803G variant occurs in isolation. The 191A variant (which defines the NAT2*14 cluster) is exclusively present in sub-Saharan Africa, and the 857A variant (which defines the NAT2*7 cluster) is mainly found in Asia and Central America. By contrast, the 590A variant occurs at roughly similar frequencies (~15–40%) in all worldwide samples, except in Amerindians where it is rare (0% and 3.7% in Ngawbe and Embera, respectively). The frequency of the 282T variant only slightly varies among human populations.

It is clearly apparent from Figures 2A and 2B that sub-Saharan African populations display greater haplotype diversity than either Europeans or Asians, and more complex relationships between *NAT2 *haplotypes are observed. A larger number of haplotypes of similar frequencies indeed occurs in these populations, generating a huge number of distinct genotypes. By contrast, in populations of Asian origin, only a few major haplotypes are found, namely NAT2*4, NAT2*6A and NAT2*7B. The mean haplotype diversity was estimated to be 0.80 ± 0.05 in Africans and 0.56 ± 0.09 in East Asians. Europeans displayed an intermediate value of 0.70 ± 0.04 [see Additional file 1].

In East Asia, the ancestral NAT2*4 haplotype associated with the rapid acetylator-phenotype accounts for more than 50% of the global variation (Thai excepted), while in Africa and Europe, the derived haplotypes associated with the slow-acetylator phenotype are predominant over NAT2*4. This observation explains the lower proportion of slow acetylators in East Asians compared to other human populations [see Additional file 2]. Derived haplotypes associated with the rapid acetylation phenotype (NAT2*12A, NAT2*13), are essentially found in Africa and are particularly frequent in Baka and Bakola Pygmies, which display a similar proportion of rapid acetylators as East Asians (83% and 90%, respectively).

#### Population structure

**Figure 3:**
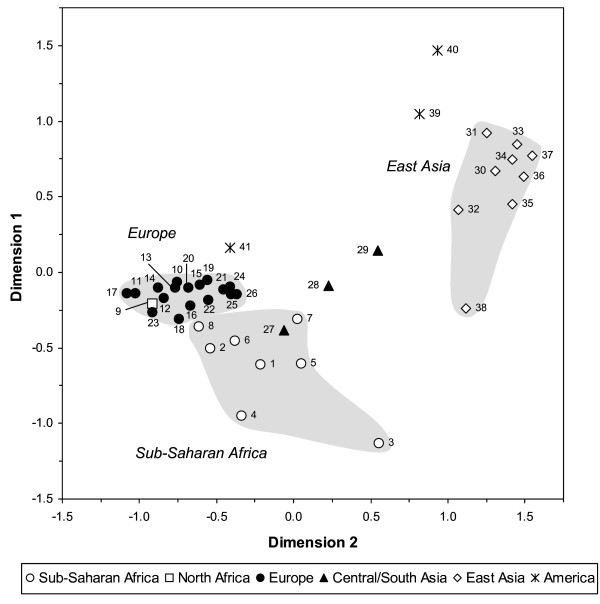
**Multidimensional scaling plot of Reynolds genetic distances among the 41 population samples of the worldwide genotyping survey.** The stress value is 0.07, indicating a very good fit of the projection to the original data. Samples are numbered according to their population code [see Additional file 1 and Additional file 2]. The shaded areas highlight the distribution of samples from sub-Saharan Africa, Europe, and East Asia in the plot.

Several differentiated clusters of populations clearly appeared in the graphical representation of the MDS (Figure 3). The sub-Saharan African populations were found relatively dispersed while Europeans and East Asians (Thai excepted) formed tight clusters with small genetic distances between pairs of populations. The proportion of nonsignificant pairwise *F*_*ST*_'s at the 5% level within each continental area was 35% and 44% in Europe and East Asia, respectively, while it was only of 11% in sub-Saharan Africa. Among Africans, Somali were genetically the most similar to Europeans. Bakola Pygmies were found highly differentiated from all other populations (except from Baka Pygmies) which could easily be explained by strong genetic drift due to isolation and small effective size. Embera and Ngawbe Amerindians did not differentiate statistically from each other; while in close proximity to East Asians, they displayed significant *F*_*ST *_values with all other samples. In contrast, Nicaraguans were found very close to Europeans, in agreement with the Amerindian-European mixed origin of the sampled individuals. Turkmen and Kyrgyz displayed *NAT2 *allele frequencies intermediary to those observed in Europeans and East Asians, which points to the continuous nature of human genetic variation worldwide [11]. The MDS plot suggests that *NAT2 *genetic differentiation patterns are related to geography, which is confirmed by a high and significant correlation coefficient (*r *= 0.47, *P *< 10^-5^) observed between genetic and geographic distances.

*F*_*ST *_gives a measure of the proportion of the genetic variance explained by differences among populations. The global *F*_*ST *_value estimated for the 41 worldwide samples was of 0.123 (*P *< 10^-5^). When grouping these 41 populations into five major geographic areas (sub-Saharan Africa, Europe/North Africa, Central/South Asia, East Asia, Central America), the vast majority of genetic variation was shown to occur within populations (83.5%), a high proportion (15%, *P *< 10^-5^) among geographic groups, and a mere 1.5% (*P *< 10^-5^) among populations within groups. When only three geographic groups were considered (35 samples grouped into sub-Saharan Africa, Europe/North Africa, and East Asia), the among groups component increased to 19%. The highest continental *F*_*ST *_value was found in sub-Saharan Africa (2.6%, *P *= 0.0001), whereas greater homogeneity was observed within East Asia (1.2%, *P *< 10^-5^), and even more so within Europe/North Africa (0.4%, *P *= 0.0002). In agreement with the MDS analysis, differentiation was very low between Europe (including Moroccans) and sub-Saharan Africa (*F*_*ST *_= 2.6%, *P *< 10^-5^), in contrast to the high *F*_*ST *_values observed between Asia and both Europe and Africa (22.2%, *P *< 10^-5^, and 23.2%, *P *< 10^-5^, respectively). Likewise, differences in the level of population differentiation were observed among the individual SNPs (Table 4). In particular, the C282T and G590A polymorphisms (defining the NAT2*6A haplotype) displayed strikingly low *F*_*ST *_values. By contrast, high *F*_*ST *_values were observed for the three SNPs T341C, C481T, and A803G which define the NAT2*5B haplotype. The highest *F*_*ST *_values at these three SNPs were observed between European and East Asian populations (around 40%).

**Table 4:** *F*_*ST *_values inferred from the 41 samples of the worldwide genotyping survey for each of the seven common SNPs of the *NAT2 *gene.

	G191A	C282T	T341C	C481T	G590A	A803G	G857A	Average *F*_*ST *_over all loci
World subdivided into								
Five geographic groups (41 populations)^a^	0.104	0.004^c^	0.257	0.231	0.022	0.241	0.061	0.152
Three geographic groups (35 populations)^b^	0.122	-0.001^c^	0.308	0.280	0.011	0.291	0.074	0.189

^a ^Sub-Saharan Africa, Europe/North Africa, Central/South Asia, East Asia, Central America [see Additional file 1 for the population composition of each geographic area]

^b ^Sub-Saharan Africa, Europe/North Africa, East Asia

^c ^Not significantly different from 0 at the 5% level

#### LD analysis

We first tested whether the amount of LD (measured as the *r*^2 ^coefficient between SNP pairs) differed between human populations. All population samples displayed similar levels of LD within each geographic area, except Somali who showed higher LD at *NAT2 *(average *r*^2 ^value = 0.589) than other sub-Saharan African samples and were, in that respect, more similar to Europeans. The mean pairwise *r*^2 ^value between the seven SNPs in the European samples (0.567 ± 0.075, including Moroccans) was significantly higher (Wilcoxon's test, *P *= 0.0002) than in both East Asians (0.276 ± 0.023) and Africans (0.243 ± 0.050, without Somali). No statistical differences in the level of LD were found between East Asian and African populations. However, the proportion of SNP pairs with *r*^2 ^≥ 0.5 was far smaller in sub-Saharan Africans (6.7%) than in both Europeans (40%) and East Asians (33.3%). Ashkenazi Jews exhibited the highest level of LD (average *r*^2 ^value = 0.763); such an excess of LD is often observed in founder populations that recently grew from relatively small sizes [12]. We then tested whether the structure of LD was similar among populations by computing the correlation between *r*^2 ^matrices of LD. Mantel's tests gave highly significant correlation values both between population pairs within geographic areas and for pairs of continental regions (*P *< 0.0001 with 10,000 permutations). Thus, although Europeans exhibited higher levels of LD at *NAT2*, the pattern of LD in this gene was found to be similar across human populations.

## Discussion

This study provides a thorough description of *NAT2 *worldwide genetic diversity. By considering only the samples adequately characterized for the seven common SNPs of the *NAT2 *gene, we avoided biases arising from incomplete genotyping studies that may lead to both allele and phenotype misclassifications. These seven SNPs are the main polymorphisms occurring in human populations at *NAT2 *and their joint analysis has been shown to be highly predictive of the acetylation phenotype with a prediction rate close to 100% [13-17]. Such a high concordance between genotype and phenotype suggests that unknown *NAT2 *variants should be present at low frequencies and therefore may not substantially influence the phenotype prediction in population studies. Although these statements are tenable in populations that have been extensively studied at *NAT2*, such as Europeans or East Asians, it is not yet known if they hold in populations poorly or inadequately studied for *NAT2 *gene variation, such as sub-Saharan African populations. As shown by Patin et al. [10] and in this study, these populations usually display a greater allelic diversity and may contain novel variants not previously reported in populations of European or Asian origin. Although two new mutations were observed in our sequencing analysis of *NAT2 *in the Mandenka sample (C345T and C638T, see Table 1), we did not disclose yet any new major polymorphism apart from the seven acknowledged ones. Consequently, only a small proportion of subjects (5%) would have been classified differently regarding their acetylator status if they had been tested for only the seven common SNPs of *NAT2 *(these 5% individuals with an unknown acetylator status would have been classified as either intermediate or rapid acetylators). A similar observation has been made for several other African populations by Patin et al. [10] (Yoruba from Nigeria, Akele Bantus from Gabon, Mbuti Pygmies from the Democratic Republic of Congo, Chagga Bantu-speakers from Tanzania, Somali and !Kung San from Namibia). However, that same survey also detected novel variants occurring at non negligible frequencies in several Pygmy populations: up to one fourth of the individuals presented an unknown acetylator status due to the high prevalence of novel mutations with an unknown functional effect. Therefore, further sequencing studies that provide information about the entire frequency spectrum rather than pre-selected variants are required to provide an unbiased description of *NAT2 *sequence variation in not yet investigated human populations.

### Genetic structure of human populations at *NAT2*

The genetic diversity patterns observed at *NAT2 *are largely consistent with those reported in many other studies of different gene regions in the human genome. The inferred levels of sequence diversity (Table 2) were found to be consistent with values reported at other highly variable human autosomal loci, such as LPL [18], GYPA [19], and CCR5 [20]. The average sequence divergence between human and chimpanzee (1.6%) was close to previous estimates of putatively neutral genomic regions [21], and the estimated *Ne *and *T*_MRCA _were also found to be in agreement with those of several other nuclear loci, which estimate the human *Ne *and *T*_MRCA _to be close to 10,000 and ~1 My, respectively [22,23]. However, one should be aware that the stochastic nature of the coalescence process used to describe the genealogy, the assumptions that have to be made (for example, the absence of recombination and selection) and the removal of data (rare recombinant haplotypes) can all have important effects on inference and lead to imprecise estimations. Sub-Saharan Africans displayed the greatest haplotype diversity at *NAT2 *and also had the largest number of unique haplotypes [see Table 3]. Furthermore, haplotypes described outside Africa were essentially a subset of the collection of *NAT2 *haplotypes found within Africa. These features of molecular diversity are generally interpreted as strong evidence for the 'Out-of-Africa' model which hypothesizes that all modern populations emerged from a common ancestral population in Africa [22]. A linear diversity gradient away from Africa was indeed observed at *NAT2 *in this study, with African populations showing the highest heterozygosities, then successively decreasing in Europeans and East Asians [see Additional file 1]. This pattern is suggestive of a gradual loss of diversity in successive colonization bottlenecks as our species grew and spread all over the world [24]. Ngawbe and Embera Amerindians displayed comparable levels of haplotype diversity to East Asians (0.42 and 0.57, respectively). However, this last observation contrasts with the recent findings of Fuselli et al. [25] that demonstrated higher *NAT2 *intra-population genetic diversity in Native Americans than in East Asians, implying more complex processes in the evolution of populations at *NAT2 *than the simple linear model exposed here above.

Another line of evidence supporting a relatively recent African origin of modern humans came from our analysis of LD patterns at *NAT2*: the lowest levels of LD were found in African populations, a common finding in empirical studies of LD in human populations. This is consistent with a larger long-term effective size of African populations and/or a bottlenecked population history of non-African populations [22,26]. Even within a small gene like *NAT2*, the SNP markers appeared to be poorly correlated in sub-Saharan Africans.

We observed a particular pattern of genetic diversity at *NAT2 *for the Thai sample compared to the other East Asian populations examined. Notably, the frequency of NAT2*4 was found to be significantly lower in Thai (0.30 *versus *around 0.50 in other Asian populations), resulting in a larger fraction of slow acetylators in this sample (50% *versus *5–20%). Interestingly, among the East Asian populations investigated, the Thai sample is the only representative of the variation of *NAT2 *in Southern East Asia. Because the full *NAT2 *gene diversity has not yet been investigated in other Southeast Asian populations, it is not possible to conclude whether this population harbors a specific profile with respect to this genetic system, or if it resembles other Southeast Asians. Further data from these latter populations are needed to speculate on a possible genetic differentiation pattern between Northern and Southern East Asian populations at the *NAT2 *locus.

### Possible selective pressures acting on *NAT2*

Because of its role in the detoxification of exogenous substances, the *NAT2 *gene has long been considered as a likely target of population-specific selective pressures. But many questions remain about the roles that population history and natural selection have played in shaping the diversity of *NAT2*. An intriguing point about this gene concerns the high frequency of poor-metabolizers and slow acetylator alleles in most human populations. This might represent the evolution of balanced polymorphisms, maintained by natural selection through heterozygote advantage or spatial-temporal selection of alternative alleles, as it has been shown for G6PD deficiencies or phenylketonuria [27,28]. An alternative explanation is that *NAT2 *may evolve under no evolutionary constraint, this enzyme being not essential from an evolutionary perspective, maybe because it is dispensable or redundant with other enzymes. In terms of the detoxification of potentially harmful environmental aromatic amines, NAT1 seems indeed to be more active than NAT2, which preempts the latter's role as a key adaptation to increase the fitness of our species [16].

In this study, we investigated whether the patterns of sequence variation at *NAT2 *were consistent with a standard, neutral equilibrium model in 13 populations of sub-Saharan Africa. All neutrality tests found no evidence of a departure from selective neutrality. But making robust inferences on the action of natural selection at a particular locus requires a thorough characterization of population history, since the neutral null hypothesis is a composite hypothesis that also makes assumptions regarding the demography of the populations. It is typically assumed that the population is in equilibrium at constant size and with no population subdivision. But suppose that African populations did pass through a relatively narrow bottleneck in the late Pleistocene and then expand. In this case, the observed data might reflect the antagonistic effects of a bottleneck (which increases the frequency of rare alleles) and balancing selection (which decreases that frequency), and the apparent evidence in favour of a neutral model of evolution would be an artefact, produced by the confounded effects of these two opposing forces. However, the results of several studies have suggested that sub-Saharan African sequence diversity was compatible with an equilibrium model of long-term constant population size and random mating [29-32]. They showed that rapid growth from a small initial size was not compatible with the African sequence data and that, if a prehistoric growth occurred, it started from a relatively large Palaeolithic population. Therefore, under such an equilibrium model, *NAT2 *can be considered as a neutrally evolving gene, at least in the sub-Saharan African populations investigated. Further studies are needed to determine whether this finding can be generalized to all African populations. It would be also useful to increase both the number of individuals studied and the size of the genomic region surveyed (by investigating for instance the entire *NAT2 *gene sequence which spans around 10 kb) to increase the power of neutrality tests which remains weak when the sample sizes and the numbers of segregating sites are small.

The global level of genetic structure (*F*_*ST *_= 0.123, *P *< 10^-5^) was found to be remarkably consistent with the average *F*_*ST *_value for the human genome [33,34], and the high correlation found between genetic and geographic distances (*r *= 0.47, *P *< 10^-5^) implies that patterns of human diversity at *NAT2 *can largely be accounted for by the simple interaction of drift and geographically-structured gene flow. However, while the overall degree of population structure at *NAT2 *was similar to previously reported values for neutral markers [35,36], unusual patterns of differentiation were observed between geographic groups. In particular, a striking differentiation of East Asia both from Europe and from Africa was found, a result that could suggest the action of region- or of population-specific selective pressures. A large variance in *F*_*ST *_values was observed for individual SNPs (Table 4), with the highest *F*_*ST *_values being observed between European and East Asian populations for the three SNPs T341C, C481T, and A803G (around 40%). These values were compared to the empirical distribution of *F*_*ST *_across a 400-kb region encompassing the *NAT *gene family (composed of the two functional *NAT1 *and *NAT2 *genes, and the *NATP *pseudogene) [see Additional file 3]. *F*_*ST *_were computed for 550 individual markers spanning the 400-kb NATs region by using publicly available HapMap SNP data (the International HapMap Project [37] /Public Release #20). They all measured population differentiation between 60 Europeans and 90 East Asians. The *F*_*ST *_values displayed by the T341C, C481T, and A803G SNP variants (pointed by a black arrow in the Additional file 3) of the *NAT2 *coding region appeared to be exceptionally large since they differed by more than three standard deviations from the mean value computed for all 550 markers in the 400-kb region (solid horizontal line in Additional file 3). Although this criterion does not have any specific statistical significance, we may expect that such exceptional values are beyond those that might be accounted for by variation at neutral loci. High *F*_*ST *_values were also observed in the 25-kb region following the *NAT2 *gene. This may be explained by the strong LD linking sites within the *NAT2 *coding exon and sites in the noncoding region next to the *NAT2 *gene, observed in both European [see Additional file 4] and East Asian populations [see Additional file 5]. Indeed, if positive natural selection was acting on *NAT2*, such as through local adaptation, it would induce hitchhiking of nearby regions [38] and would increase interpopulation differentiation at linked neutral sites, as distinct haplotypes would be fixed in different populations. Besides, it is also interesting to note the high *F*_*ST *_values in the region surrounding the *NAT1 *gene and, surprisingly, in a noncoding segment preceding the *NATP *pseudogene.

By contrasting the *F*_*ST *_of individual SNPs to the empirical distribution of *F*_*ST *_across a genomic region, it is possible to identify those loci that exhibit unusual patterns of population differentiation as potential candidates for the action of natural selection [39,40]. Several studies have used this strategy to detect the action of selection on specific genes or on a genome-wide scale [33,34,41]. However, genome-wide surveys of *F*_*ST *_have demonstrated substantial variation of *F*_*ST *_values across the genome, even among SNPs that are very close to each other [33,34], thus stressing the difficulty to distinguish between non-random events such as local selection and random events such as extreme genetic drift as the agents responsible for the unusual patterns observed. Individual-marker *F*_*ST *_estimates are probably too variable to be reliable indicators of past selective events and other more powerful tests are required to provide unambiguous evidence of natural selection. The availability of genotype data for additional markers surrounding the *NAT2 *gene would enable the implementation of the long-range haplotype test [42] which has better power for identifying signatures of recent positive selection.

A possible explanation for the unusually large difference in allele frequencies observed between European and East Asian populations for the *NAT2 *variants could be the impact of population-specific selective pressures. The main molecular basis for the high discrepancy between Europeans and East Asians is that the most common allele at the *NAT2 *locus in Europeans (NAT2*5B) is very rare in East Asians and could represent a different selective advantage within the gene pools of these separate populations. Patin et al. [43] found evidence of a rapid increase in frequency of the NAT2*5B haplotype in Western and Central Eurasian populations in the last ~6,500 years in response to positive selection, suggesting that this slow allele probably conferred some selective advantage to its carriers in this part of the world. A thorough survey of *NAT2 *sequence variation in East Asians will be necessary to determine whether the predominance of the rapid-acetylator NAT2*4 allele over the slow ones is the result of local positive selection or whether it can be explained by stochastic processes such as genetic drift.

## Conclusion

This study provides a thorough description of the worldwide haplotype diversity and LD structure of the *NAT2 *gene. We found that patterns of *NAT2 *sequence variation are consistent with selective neutrality in all sub-Saharan African populations investigated, whereas the high level of population differentiation between Europeans and East Asians inferred from SNPs may suggest population-specific selective pressures acting at this locus, probably caused by differences in diet or exposure to other environmental signals.

## Methods

### *NAT2* sequencing of the Mandenka

Full sequence diversity of *NAT2 *exon 2, which contains the entire protein-coding region, was determined in 97 healthy unrelated individuals (62 men, 35 women) from the Niokholo Mandenka. This agriculturalist population from Eastern Senegal speaks a language belonging to Mande, a major primary branch of the Niger-Congo language family. We used DNA extracted from the lymphoblastoid cell lines (LCL) described in Excoffier et al. [44]. The sample size considered was sufficient to detect *NAT2 *variants present at a frequency ≥ 3%, with a probability of at least 99%.

A 1,211-bp fragment covering the entire coding region of *NAT2 *was amplified by PCR and subsequently sequenced on both DNA strands on an ABI 3730xl automated sequencer (Applied Biosystems) using three internal primer pairs, as described in Cascorbi et al. [45] (sequencing service provided by Macrogen, Seoul, Korea). The sequence variation of a 1188-bp fragment (from nt -59 to nt +1129) was fully surveyed, which includes the 870 bp of the *NAT2 *coding exon and 318 bp of non-coding flanking parts. All singletons were verified by PCR reamplification and resequencing the PCR products in both directions. The observed genotype frequencies at all polymorphic sites were in conformity with the assumptions of Hardy-Weinberg equilibrium when tested using Fisher's exact tests. Haplotype reconstruction was performed using the Bayesian method implemented in PHASE v.2.1 [46]. All 194 haplotype sequences were submitted to GenBank [GenBank:DQ904040-DQ904233].

For comparison purposes, we merged the *NAT2 *sequence data set with a collection of previously published sequences of the *NAT2 *coding exon (870 bp) [10]. This additional dataset consists in 285 individuals from 12 sub-Saharan African populations, including Yoruba from Nigeria (N = 31); Baka Pygmies (N = 31), Bakola Pygmies (N = 26), Bedzan Pygmies (N = 32) and Ngumba Bantus (N = 16) from Cameroon; Baka Pygmies (N = 16) and Akele Bantus (N = 26) from Gabon; Biaka Pygmies from Central African Republic (N = 24); Mbuti Pygmies from the Democratic Republic of Congo (N = 24); Chagga Bantu-speakers from Tanzania (N = 32); Somali (N = 20) and !Kung San from Namibia (N = 7).

### *NAT2* worldwide genotyping survey

We selected from published reports up to 2006 all the population samples that were genotyped for the seven most common SNPs at *NAT2 *and for which genotype data was available. Among these seven SNPs, all located in the coding exon, four result in an amino acid substitution that leads to a significant decrease in acetylation capacity (G191A, T341C, G590A, G857A). The other three are either silent mutations (C282T, C481T) or a non-synonymous substitution that does not alter the phenotype (A803G).

A complete list of the selected samples is provided in Additional file 1, along with a full description of each sample and references. The collected data consisted of 6,727 individuals (13,454 chromosomes) from 41 human populations representing major geographic regions: Europe (17 samples), North Africa (1), sub-Saharan Africa (8), Central/South Asia (3), East Asia (9), and Central America (3). The geographical distribution of the samples is shown in Additional file 2. Sample sizes range from 24 (Somali) to 1000 (Korean) individuals, with an average of 160 individuals per sample. For 13 samples, genotype data at one SNP (11 samples) or two SNPs (2 samples) out of the seven were missing. It involves either G191A (which has been shown to be extremely rare in Europeans and Asians) or the synonymous C282T polymorphism. These samples were excluded from LD analyses.

### Statistical analyses

All sequence analyses were performed on both the Mandenka sample and on each of the 12 African samples of Patin et al. [10]. Homologous sequences from one chimpanzee (*Pan troglodytes*; Ensembl Chinpanzee genome) and one rhesus monkey (*Macaca mulatta*; GenBank XM_001098734) were used to infer SNPs' ancestral state. Each of these also served as an outgroup for evolutionary and population genetic tests.

DnaSP v.4.10 [47] was used to compute, in each sample, the nucleotide (*π*) and haplotype (*H*) diversity, Watterson's *θ*_*w *_[48], as well as to perform several neutrality tests to detect signals of natural selection: Tajima's *D *[49], Fu and Li's F* and D* [50], Fu's *F*_*s *_[51], and Fay and Wu's *H *[52]. The statistical significance of the tests was estimated from 10,000 coalescent simulations of an infinite site locus, conditional on sample size, both with and without recombination. The McDonald-Kreitman test [53] was applied to detect deviation from the neutral expectation of equal rates of nonsynonymous to synonymous polymorphism within humans and nonsynonymous to synonymous fixed substitutions between humans and chimpanzee.

A coalescence model for the ancestral history of a sample of genes was used to estimate the time scale of polymorphic variation in the *NAT2 *gene. The time to the most recent common ancestor (*T*_MRCA_) and mutation ages were estimated from the *NAT2 *gene tree, conditional on a maximum-likelihood estimate of *θ*(${\hat{\theta }}_{ML}$), the population mutation parameter. These estimates were computed with GeneTree v.9.0 [54] (running 10^6 ^replications), under a standard coalescence model assuming neutrality, the infinite-sites mutation model (haplotypes presumably affected by recurrent mutation or recombination were removed from the analysis), random mating, and constant population size. All estimates were inferred on individual population samples, to avoid biases due to population structure. Time, scaled in 2*Ne *units, was converted into years by use of a 25-year generation time and the value of the effective population size (*Ne*) obtained as ${\hat{\theta }}_{ML}$ divided by 4 *μ*. The neutral mutation rate per gene per generation (*μ*) was estimated based on human-chimpanzee sequence divergence, assuming a divergence time of 5 million years (My) [55].

For the worldwide genotyping survey, we inferred *NAT2 *haplotypes from the unphased multi-locus genotypes using PHASE v.2.1 software [46]. The individual acetylation phenotypes were then predicted from the haplotype combination at *NAT2*, in accordance with the acknowledged classification of NAT2* alleles based on their functional impact [see Table 3]: individuals with two low activity alleles were classified as slow acetylators, those with two functional alleles as rapid acetylators, and those with both a slow and a functional allele as intermediate acetylators.

Median-joining networks [56] describing the mutational relationships among the inferred *NAT2 *haplotypes were generated using Network 4.1.1 software [57].

Population structure in the worldwide genotyping survey set was investigated by an analysis of molecular variance (AMOVA) [58] that included the molecular distance matrix among *NAT2 *haplotypes. Population differentiation was tested by permutation tests (20,000 permutations) based on the *F*_*ST *_statistic. Coancestry coefficients, or linearized *F*_*ST *_values [59], were computed among populations and the resulting genetic distance matrix was used for multidimensional scaling analysis (MDS) [60] performed with the NTSYS v.2.1 software [61]. A Mantel test was applied to test the correlation of pairwise genetic distances with geographic distances, computed as great-circle distances between populations from their coordinates of latitude and longitude (US Caucasians and Ashkenazi Jews were excluded from the analysis since they could not be allocated precisely to a specific area). All calculations, including random-permutation procedures to assess statistical significance, were performed by use of the Arlequin v.3.0 package [62].

Pairwise LD between the seven genotyped SNPs was estimated by computing the *r*^2 ^statistic [63] with DnaSP [47], after the exclusion, in each population of the worldwide genotyping survey, of SNPs with minor allele frequency (MAF) < 0.05. Statistical significance of LD between SNP pairs was assessed using Fisher's exact tests followed by Bonferroni corrections. Mantel tests to compare *r*^2 ^matrices were performed using the program CADM [64]. Comparisons were made between populations within each continental group. Subsequently, *r*^2 ^values were recalculated for populations pooled into geographical groups and Mantel tests were applied.

## Authors' contributions

AS conceived of the study, collected data, performed the majority of the statistical analyses and wrote the initial draft of the manuscript. AL and PD participated to study design, supervised analyses, and were involved in drafting the manuscript. AL and EP provided the Mandenka sample. NG and RK participated in the *NAT2 *gene sequencing in the Mandenka sample. EP provided the conceptual framework for the study, supervised the statistical analyses and finalized the manuscript. All authors read and approved the final manuscript.

## Supplementary Material

Additional file 1 Summary description of the 41 samples included in the worldwide genotyping survey. Click here for file

Additional file 2 Distribution of *NAT2* predicted acetylation phenotypes in the 41 samples of the worldwide genotyping survey. Click here for file

Additional file 3 Distribution of *F_ST _*values between Europeans and East Asians across a 400-kb segment encompassing the human *NAT* gene family on chromosome 8. Click here for file

Additional file 4 LD/block structure of the European HapMap sample across a 400-kb segment encompassing the human *NAT* gene family on chromosome 8. Click here for file

Additional file 5 LD/block structure of the East Asian HapMap sample across a 400-kb segment encompassing the human *NAT* gene family on chromosome 8. Click here for file
