# Supplementary material for: Worldwide distribution of NAT2 diversity: Implications for NAT2 evolutionary history
Source: BMC Genet. 2008 Feb 27;9:21. doi: 10.1186/1471-2156-9-21 (PMC2292740; doi:10.1186/1471-2156-9-21)
Supplement: Additional file 1 — Summary description of the 41 samples included in the worldwide genotyping survey. [file 1471-2156-9-21-S1.doc]

**Additional file 1: Summary description of the 41 samples included in the worldwide genotyping survey**

**Additional file 1** (Continued)

**Additional file 1** (Continued)

^a^ Population code number

^b^ Number of inferred haplotypes in the sample

^c^ Haplotype diversity

^d^ C282T missing

^e^ G191A and C282T missing

^f^ G191A missing

^g^ The frequencies of *NAT2* haplotypes determined in this study on patients with rheumatoid arthritis were similar to those determined in Japanese controls

**References**

1. Loktionov A, Moore W, Spencer SP, Vorster H, Nell T, O'Neill IK, Bingham SA, Cummings JH (2002) Differences in N-acetylation genotypes between Caucasians and Black South Africans: implications for cancer prevention. Cancer Detect Prev 26:15-22.

2. Patin E, Harmant C, Kidd KK, Kidd J, Froment A, Mehdi SQ, Sica L, Heyer E, Quintana-Murci L (2006) Sub-Saharan African coding sequence variation and haplotype diversity at the NAT2 gene. Hum Mutat 27:720.

3. The International HapMap Consortium (2003) The International HapMap Project. Nature 426:789-796.

4. This study

5. Delomenie C, Sica L, Grant DM, Krishnamoorthy R, Dupret JM (1996) Genotyping of the polymorphic N-acetyltransferase (NAT2*) gene locus in two native African populations. Pharmacogenetics 6:177-185.

6. Agundez JA, Olivera M, Ladero JM, Rodriguez-Lescure A, Ledesma MC, Diaz-Rubio M, Meyer UA, Benitez J (1996) Increased risk for hepatocellular carcinoma in NAT2-slow acetylators and CYP2D6-rapid metabolizers. Pharmacogenetics 6:501-512.

7. Krajinovic M, Richer C, Sinnett H, Labuda D, Sinnett D (2000) Genetic polymorphisms of N-acetyltransferases 1 and 2 and gene-gene interaction in the susceptibility to childhood acute lymphoblastic leukemia. Cancer Epidemiol Biomarkers Prev 9:557-562.

8. Deitz AC, Zheng W, Leff MA, Gross M, Wen WQ, Doll MA, Xiao GH, Folsom AR, Hein DW (2000) N-Acetyltransferase-2 genetic polymorphism, well-done meat intake, and breast cancer risk among postmenopausal women. Cancer Epidemiol Biomarkers Prev 9:905-910.

9. Cascorbi I, Drakoulis N, Brockmoller J, Maurer A, Sperling K, Roots I (1995) Arylamine N-acetyltransferase (NAT2) mutations and their allelic linkage in unrelated Caucasian individuals: correlation with phenotypic activity. Am J Hum Genet 57:581-592.

10. Schnakenberg E, Lustig M, Breuer R, Werdin R, Hubotter R, Dreikorn K, Schloot W (2000) Gender-specific effects of NAT2 and GSTM1 in bladder cancer. Clin Genet 57:270-277.

11. Mrozikiewicz PM, Cascorbi I, Brockmoller J, Roots I (1996) Determination and allelic allocation of seven nucleotide transitions within the arylamine N-acetyltransferase gene in the Polish population. Clin Pharmacol Ther 59:376-382.

12. Habalova V, Salagovic J, Kalina I, Stubna J (2005) A pilot study testing the genetic polymorphism of N-acetyltransferase 2 as a risk factor in lung cancer. Neoplasma 52:364-368.

13. Rabstein S, Unfried K, Ranft U, Illig T, Kolz M, Rihs HP, Mambetova C, Vlad M, Bruning T, Pesch B (2006) Variation of the N-acetyltransferase 2 gene in a Romanian and a Kyrgyz population. Cancer Epidemiol Biomarkers Prev 15:138-141.

14. Gaikovitch EA, Cascorbi I, Mrozikiewicz PM, Brockmoller J, Frotschl R, Kopke K, Gerloff T, Chernov JN, Roots I (2003) Polymorphisms of drug-metabolizing enzymes CYP2C9, CYP2C19, CYP2D6, CYP1A1, NAT2 and of P-glycoprotein in a Russian population. Eur J Clin Pharmacol 59:303-312.

15. Aynacioglu AS, Cascorbi I, Mrozikiewicz PM, Roots I (1997) Arylamine N-acetyltransferase (NAT2) genotypes in a Turkish population. Pharmacogenetics 7:327-331.

16. Guo WC, Lin GF, Zha YL, Lou KJ, Ma QW, Shen JH (2004) N-Acetyltransferase 2 gene polymorphism in a group of senile dementia patients in Shanghai suburb. Acta Pharmacol Sin 25:1112-1117.

17. Tanaka E, Taniguchi A, Urano W, Nakajima H, Matsuda Y, Kitamura Y, Saito M, Yamanaka H, Saito T, Kamatani N (2002) Adverse effects of sulfasalazine in patients with rheumatoid arthritis are associated with diplotype configuration at the N-acetyltransferase 2 gene. J Rheumatol 29:2492-2499.

18. Deguchi M, Yoshida S, Kennedy S, Ohara N, Motoyama S, Maruo T (2005) Lack of association between endometriosis and N-acetyl transferase 1 (NAT1) and 2 (NAT2) polymorphisms in a Japanese population. J Soc Gynecol Investig 12:208-213.

19. Lee KM, Park SK, Kim SU, Doll MA, Yoo KY, Ahn SH, Noh DY, Hirvonen A, Hein DW, Kang D (2003) N-acetyltransferase (NAT1, NAT2) and glutathione S-transferase (GSTM1, GSTT1) polymorphisms in breast cancer. Cancer Lett 196:179-186.

20. Lee SY, Lee KA, Ki CS, Kwon OJ, Kim HJ, Chung MP, Suh GY, Kim JW (2002) Complete sequencing of a genetic polymorphism in NAT2 in the Korean population. Clin Chem 48:775-777.

21. Jorge-Nebert LF, Eichelbaum M, Griese EU, Inaba T, Arias TD (2002) Analysis of six SNPs of NAT2 in Ngawbe and Embera Amerindians of Panama and determination of the Embera acetylation phenotype using caffeine. Pharmacogenetics 12:39-48.

22. Martinez C, Agundez JA, Olivera M, Llerena A, Ramirez R, Hernandez M, Benitez J (1998) Influence of genetic admixture on polymorphisms of drug-metabolizing enzymes: analyses of mutations on NAT2 and C gamma P2E1 genes in a mixed Hispanic population. Clin Pharmacol Ther 63:623-628.
