# Supplementary material for: Worldwide distribution of NAT2 diversity: Implications for NAT2 evolutionary history
Source: BMC Genet. 2008 Feb 27;9:21. doi: 10.1186/1471-2156-9-21 (PMC2292740; doi:10.1186/1471-2156-9-21)
Supplement: Additional file 2 — Distribution of NAT2 predicted acetylation phenotypes in the 41 samples of the worldwide genotyping survey. [file 1471-2156-9-21-S2.doc]

**Additional file 2: Distribution of *NAT2* predicted acetylation phenotypes in the 41 samples of the worldwide genotyping survey**

**
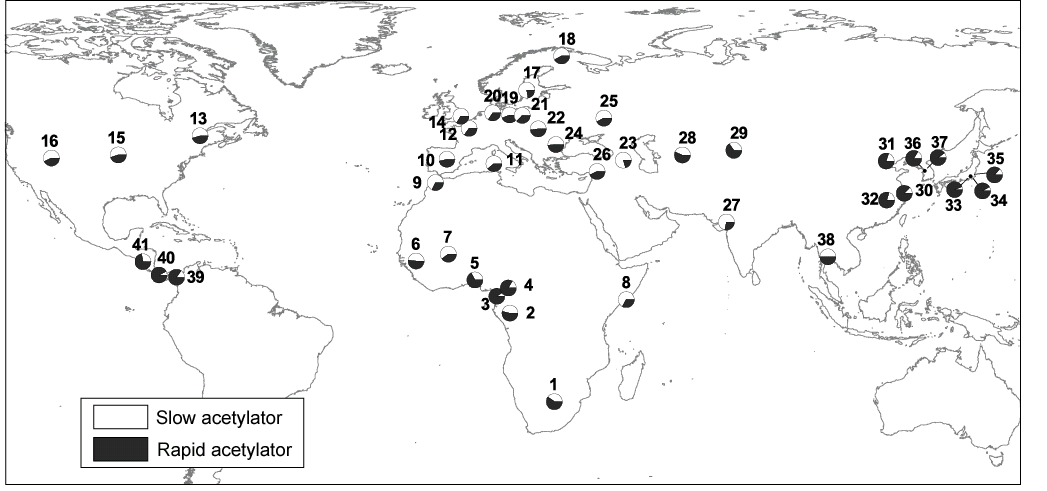
**

Each pie chart reports the proportion of rapid and slow acetylators in a sample. Intermediate acetylators were included into the rapid-acetylator phenotype. Samples are numbered according to their population code [see Additional file 1]: (1) 101 Tswana, (2) 50 Ateke Bantus, (3) 40 Bakola Pygmies, (4) 30 Baka Pygmies, (5) 60 Yoruba, (6) 97 Mandenka, (7) 50 Dogons, (8) 24 Somali, (9) 44 Moroccans, (10) 258 Spanish, (11) 49 Sardinians, (12) 60 French, (13) 291 French-Canadians, (14) 112 UK Caucasians, (15) 387 US Caucasians, (16) 60 US Caucasians, (17) 50 Swedes, (18) 48 Saami, (19) 844 Germans, (20) 223 Germans, (21) 248 Polish, (22) 167 Slovaks, (23) 40 Ashkenazi Jews, (24) 140 Romanians, (25) 290 Russians, (26) 303 Turks, (27) 50 Gujarati, (28) 50 Turkmen, (29) 290 Kyrgyz, (30) 112 Han Chinese, (31) 45 Han Chinese, (32) 44 Chinese, (33) 144 Japanese, (34) 172 Japanese, (35) 44 Japanese, (36) 288 Koreans, (37) 1,000 Koreans, (38) 44 Thai, (39) 136 Embera, (40) 105 Ngawbe, (41) 137 Nicaraguans.
