# Supplementary material for: Worldwide distribution of NAT2 diversity: Implications for NAT2 evolutionary history
Source: BMC Genet. 2008 Feb 27;9:21. doi: 10.1186/1471-2156-9-21 (PMC2292740; doi:10.1186/1471-2156-9-21)
Supplement: Additional file 3 — Distribution of FST values between Europeans and East Asians across a 400-kb segment encompassing the human NAT gene family on chromosome 8. [file 1471-2156-9-21-S3.doc]

**Additional file 3: Distribution of *F_ST_* values between Europeans and East Asians across a 400-kb segment encompassing the human *NAT* gene family on chromosome 8**

Available SNP data from the International HapMap Project [37] (Public Release #20) was used to compute *F_ST_* values between 60 individuals of European origin from Utah (population code 16 in this paper) and 90 East Asians, pooling 45 Han Chinese from Beijing and 45 Japanese from Tokyo (population codes 31 and 35, respectively). Only those SNPs that were genotyped in all population samples and with MAF ≥ 5% in at least one sample were selected. A total of 550 SNP markers were used that span a 377-kb region (distance between the first and the last marker), with a mean distance between markers of ~ 680 bp. The average *F_ST_* for all SNPs across the 400-kb region is shown as a horizontal solid line. The dashed line provides a guide for identifying exceptionally high values (i.e., values differing by more than three standard deviations from the 400-kb mean value). The location of the *NAT1*, *NATP* and *NAT2* genes within the 400-kb surveyed region is shown below the plot. The *NAT2* coding exon is shown as a black box within the *NAT2* gene. The black arrow points to the *F_ST_* values displayed by the three T341C, C481T, and A803G SNP variants.
