# Supplementary material for: Worldwide distribution of NAT2 diversity: Implications for NAT2 evolutionary history
Source: BMC Genet. 2008 Feb 27;9:21. doi: 10.1186/1471-2156-9-21 (PMC2292740; doi:10.1186/1471-2156-9-21)

**Additional file 5:** LD/block structure of the East Asian HapMap sample (90 individuals composed of 45 Han Chinese from Beijing and 45 Japanese from Tokyo; population codes 31 and 35, respectively) across a 400-kb segment encompassing the human *NAT* gene family on chromosome 8. Using SNP data from the International HapMap Project [37] (Public Release #20), pairwise D’ values among common SNPs (MAF>5%) were computed with the Haploview program. Each square represents the magnitude of linkage disequilibrium for a single pair of markers, with red color indicating strong (D’ > 0.8) and statistically significant linkage disequilibrium (logarithm of odds >2.0).


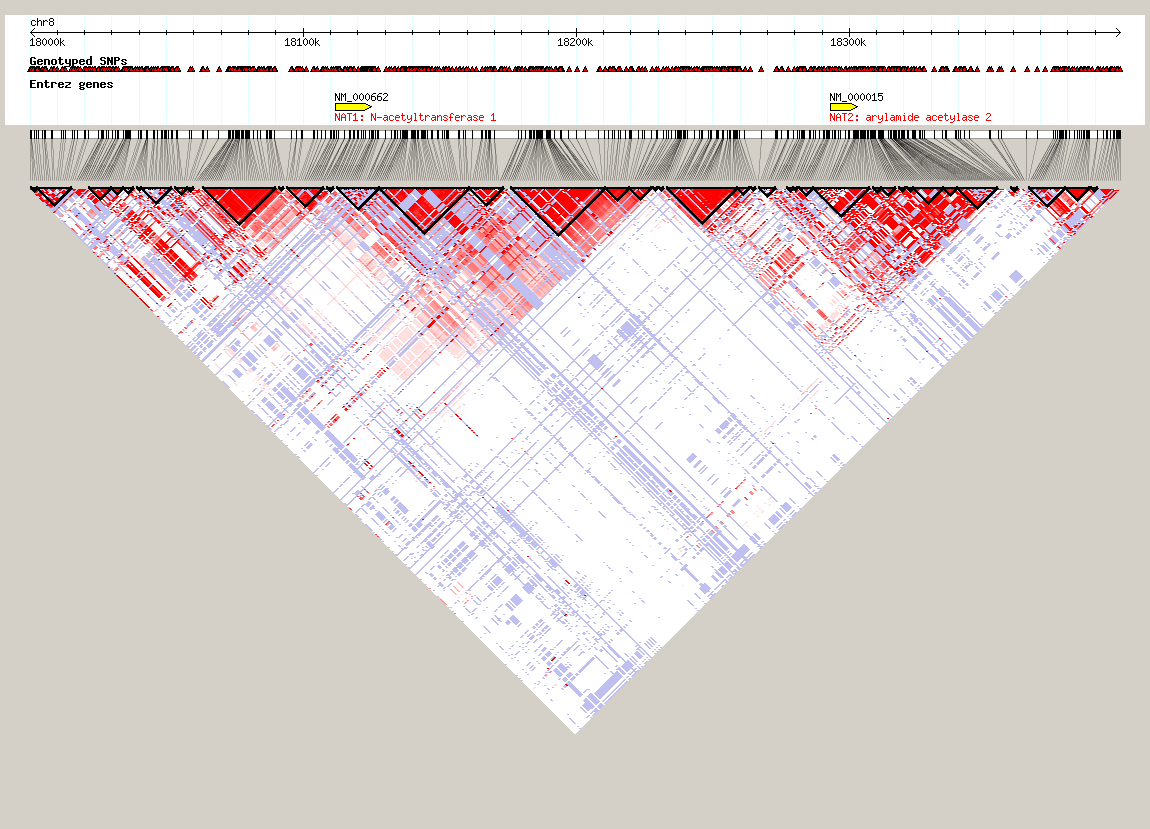

Supplement: Additional file 5 — LD/block structure of the East Asian HapMap sample across a 400-kb segment encompassing the human NAT gene family on chromosome 8. [file 1471-2156-9-21-S5.doc]
